# Genetic modification of *PIN* genes induces causal mechanisms of stay-green drought adaptation phenotype

**DOI:** 10.1093/jxb/erac336

**Published:** 2022-08-13

**Authors:** Andrew K Borrell, Albert C S Wong, Barbara George-Jaeggli, Erik J van Oosterom, Emma S Mace, Ian D Godwin, Guoquan Liu, John E Mullet, Patricia E Klein, Graeme L Hammer, Greg McLean, Colleen Hunt, David R Jordan

**Affiliations:** University of Queensland, Queensland Alliance for Agriculture and Food Innovation (QAAFI), Warwick, QLD 4370, Australia; University of Queensland, QAAFI, Brisbane, QLD 4072, Australia; University of Queensland, Queensland Alliance for Agriculture and Food Innovation (QAAFI), Warwick, QLD 4370, Australia; Agri-Science Queensland, Department of Agriculture & Fisheries, Warwick, QLD 4370, Australia; University of Queensland, QAAFI, Brisbane, QLD 4072, Australia; University of Queensland, Queensland Alliance for Agriculture and Food Innovation (QAAFI), Warwick, QLD 4370, Australia; Agri-Science Queensland, Department of Agriculture & Fisheries, Warwick, QLD 4370, Australia; University of Queensland, QAAFI, Brisbane, QLD 4072, Australia; University of Queensland, QAAFI, Brisbane, QLD 4072, Australia; Department of Biochemistry and Biophysics, Texas A&M University, College Station, TX 77843, USA; Department of Horticultural Sciences, Texas A&M University, College Station, TX 77843, USA; University of Queensland, QAAFI, Brisbane, QLD 4072, Australia; University of Queensland, QAAFI, Brisbane, QLD 4072, Australia; Agri-Science Queensland, Department of Agriculture & Fisheries, Warwick, QLD 4370, Australia; University of Queensland, Queensland Alliance for Agriculture and Food Innovation (QAAFI), Warwick, QLD 4370, Australia

**Keywords:** Canopy development, cereals, drought adaptation, *PIN* genes, root architecture, stay-green

## Abstract

The stay-green trait is recognized as a key drought adaptation mechanism in cereals worldwide. Stay-green sorghum plants exhibit delayed senescence of leaves and stems, leading to prolonged growth, a reduced risk of lodging, and higher grain yield under end-of-season drought stress. More than 45 quantitative trait loci (QTL) associated with stay-green have been identified, including two major QTL (*Stg1* and *Stg2*). However, the contributing genes that regulate functional stay-green are not known. Here we show that the *PIN FORMED* family of auxin efflux carrier genes induce some of the causal mechanisms driving the stay-green phenotype in sorghum, with *SbPIN4* and *SbPIN2* located in *Stg1* and *Stg2*, respectively. We found that nine of 11 sorghum *PIN* genes aligned with known stay-green QTL. In transgenic studies, we demonstrated that *PIN* genes located within the *Stg1* (*SbPIN4*), *Stg2* (*SbPIN2*), and *Stg3b* (*SbPIN1*) QTL regions acted pleiotropically to modulate canopy development, root architecture, and panicle growth in sorghum, with *SbPIN1*, *SbPIN2*, and *SbPIN4* differentially expressed in various organs relative to the non-stay-green control. The emergent consequence of such modifications in canopy and root architecture is a stay-green phenotype. Crop simulation modelling shows that the *SbPIN2* phenotype can increase grain yield under drought.

## Teaser

Genetic modification of *PIN*genes modulated canopy development and root architecture in sorghum, inducing causal mechanisms of the stay-green drought adaptation phenotype.

## Introduction

One of the most significant challenges facing crop improvement programmes globally is that of matching crop production with the dietary needs of the human population in highly variable environments, thereby ensuring food security in the face of climate change. Drought caused global economic losses in agriculture of ~US $29 billion over the past decade (FAO, 2018). The frequency and intensity of severe water scarcity are expected to increase due to global warming, thereby negatively affecting grain yield of rain-fed crops (e.g. wheat, sorghum, and barley), which are a key source of calories and protein for humans (Trnka *et al.*, 2019). A global scarcity of water resources will necessitate the development of climate-resilient crops that use water more efficiently (Varshney *et al.*, 2021). To meet these challenges, genetic resources must be better assessed (or alternatively modified by technologies such as gene editing) to identify molecular mechanisms that enhance developmental plasticity in traits regulating water supply and demand, thereby enabling sessile plants to adapt more readily to a hotter and drier world.

Due to its prevalence and origin in the more arid areas of the world, sorghum (*Sorghum bicolor* L. Moench), a C_4_ cereal that provides staple food for millions of the most food-insecure people in Asia and Africa (Hariprasanna and Patil, 2015), is a repository of cereal drought adaptation mechanisms (Borrell *et al.*, 2021). It is particularly well known for the so-called ‘stay-green’ trait, which enables plants to retain green leaves and stems, and therefore maintain growth for longer during end-of-season droughts (Borrell *et al.*, 2000b, 2014a). This is associated with increased grain yield, but also with better lodging resistance compared with plants without the stay-green trait, which senesce more quickly (Rosenow, 1977; Jordan *et al.*, 2012).

**Fig. 1.**
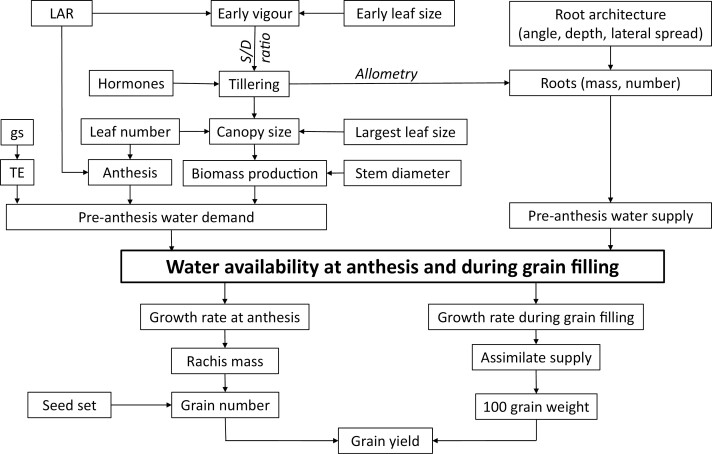
Flowchart of crop physiological processes that determine plant size and crop water use of sorghum during early-, mid-, and late-season growth. Water availability at anthesis is critical to grain yield development. gs, stomatal conductance; LAR, leaf appearance rate; S/D ratio, supply/demand ratio; TE, transpiration efficiency.

More than 45 major and minor quantitative trait loci (QTL) contributing to the stay-green (*Stg*) phenotype have been identified (Crasta *et al.*, 1999; Subudhi *et al.*, 2000; Tao *et al.*, 2000; Xu *et al.*, 2000; Kebede *et al.*, 2001; Haussmann *et al.*, 2002; Srinivas *et al.*, 2009; Sabadin *et al.*, 2012; Rama Reddy *et al.*, 2014; Wang *et al.*, 2014; Sukumaran *et al.*, 2016). In recent years, our understanding of the nature of stay-green in sorghum has moved from perceiving it as a relatively simple trait to seeing it as a complex phenotype that is the result of a range of component traits that influence senescence directly but, more importantly, indirectly through their impacts on water supply and demand. Using near-isogenic lines containing some of these major QTL (*Stg1–Stg4*), we have previously shown that the stay-green phenotype results from above- or below-ground plant architecture changes and physiological differences that increase water availability during periods most critical to yield, such as flowering and grain filling. This may be achieved by (i) increasing water supply via the modification of root architecture (Mace *et al.*, 2012); (ii) decreasing water demand via reduced canopy size early in crop development and hence preserving subsoil water supplies for uptake during grain fill (Borrell *et al.*, 2000a, 2014b); (iii) using water more efficiently via enhanced transpiration efficiency (Vadez *et al.*, 2011); or (iv) by combinations of all three mechanisms (Fig. 1). Such adaptations, exhibited by plants containing the stay-green trait, can significantly improve yield stability in agriculture.

In cereals, canopy size is determined by the total green leaf area of all mainstem leaves, plus the combined leaf area of the secondary branches, or tillers. Near-isogenic lines containing stay-green QTL can have smaller upper mainstem leaves and/or reduced tiller number (Borrell *et al.*, 2014a, b). The majority of tillers in sorghum are axillary basal tillers, which grow in the leaf axils of lower mainstem leaves. While each mainstem leaf is capable of bearing an axillary tiller, the outgrowth of tiller buds is thought to depend on the supply of assimilates (namely sucrose) from source leaves (nutritive theory), but also on the ability of the bud to export auxin into the polar auxin transport stream (canalization theory) (Sauer *et al.*, 2006; Mason *et al.*, 2014). Both of these processes may be affected by environmental and internal cues, such as the availability of water, light, carbon, and nutrients (Leyser, 2010; Alam *et al.*, 2014; Wang *et al.*, 2018).

Auxin export from axillary buds is dependent on PIN-FORMED (PIN) proteins (Prusinkiewicz *et al.*, 2009), which are a family of secondary transporters that act as efflux carriers of the plant signal molecule auxin from cells and play an important role in multiple developmental processes by regulating asymmetric auxin distribution (Křeček *et al.*, 2009). The similarity among PIN proteins suggests that they have diverged from a single ancestral sequence. Interestingly, monocots have higher *PIN* gene diversity than dicots, with three to four homologues for every Arabidopsis *PIN* and some monocot-specific *PIN* genes identified (Křeček *et al.*, 2009; Shen *et al.*, 2010). *PIN* genes control growth and patterning in roots and leaves (Blilou *et al.*, 2005), including phyllotaxis (Reinhardt *et al.*, 2003). They mediate gravitropism in Arabidopsis roots (Abas *et al.*, 2006) and are important in the formation of vascular bundles and polar auxin transport (Friml *et al.*, 2002). It has recently been shown in Arabidopsis that local modulation of the auxin pathway can result in distinct root system architectures (RSAs) that can be adaptive in specific environments (Ogura *et al.*, 2019; Pandey and Bennett, 2019). In these studies, EXOCYST70A3 was identified as a modulator of auxin, causing variation in RSA by acting on PIN4 protein distribution, leading to altered root gravitropic responses. Furthermore, overexpression of *OsPIN2* in rice (*Oryza sativa* L.) leads to increased tiller number and less erect leaves (Chen *et al.*, 2012), and *OsPIN1* affects adventitious root emergence and tillering in rice (Xu *et al.*, 2005).

The objective of this study was to show how the genetic modification of *PIN* genes can induce causal mechanisms of the stay-green drought adaptation phenotype in sorghum. Here we show that nine of the 11 members of the *PIN* family of auxin efflux carrier genes in sorghum co-locate with *Stg* QTL and that transforming a non-stay-green sorghum genotype with *PIN* genes located within the major *Stg* QTL, *Stg1* (*SbPIN4*), *Stg2* (*SbPIN2*), and *Stg3b* (*SbPIN1*) chromosomal regions leads to changes in plant architecture that can potentially modify canopy water supply and demand in such a way that a stay-green phenotype becomes an emergent consequence. Using APSIM, a bio-physiological crop growth model, we also show that the observed effects of the *SbPIN2* gene on crop canopy architecture probably lead to substantial yield increases and production risk reductions in the water-limited environments typical of many global sorghum-growing areas. This is the first time *PIN* genes have been linked to changes in plant architecture known to lead to increased drought adaptation by regulating the causal mechanisms of the stay-green phenotype in a major cereal.

## Materials and methods

### *Co-location of* PIN *genes within stay-green QTL*

A total of 193 stay-green QTL identified in 11 previously published QTL studies (Crasta *et al.*, 1999; Subudhi *et al.*, 2000; Tao *et al.*, 2000; Xu *et al.*, 2000; Kebede *et al.*, 2001; Haussmann *et al.*, 2002; Srinivas *et al.*, 2009; Sabadin *et al.*, 2012; Rama Reddy *et al.*, 2014; Wang *et al.*, 2014; Sukumaran *et al.*, 2016) were extracted from the sorghum QTL atlas, with confidence intervals projected onto the sorghum consensus map as defined in Mace *et al.* (2019). *PIN* gene enrichment in the regions of the genome containing stay-green QTL was determined through χ^2^ analyses.

### *Transformation of* SbPIN1, SbPIN2, *and* SbPIN4 *into sorghum*

Three plasmids, namely *SbPIN1*, *SbPIN2*, and *SbPIN4*, were designed. The native promoter used for each gene was 1.2 kb of upstream sequence. The 5ʹ-untranslated region (UTR), 3ʹ-UTR, and all CDSs (coding sequences) were identified and selected, in addition to the NOS (*nopaline synthase*) terminator. After that the whole sequence of each gene was synthesized and cloned into the pUC57-Amp vector (Synbio Technologies, Genes for Life). A highly efficient micro projectile transformation system for sorghum (*Sorghum bicolor* L.) was performed as described by Liu and Godwin (2012). Briefly, immature embryos (IEs) of inbred line Tx430 were used. Plant material preparation, media for tissue culture, and embryo isolation were carried out as described by Liu and Godwin (2012). Co-bombardment was deployed with the neomycin phosphotransferase II (*nptII*) selective gene driven by the maize ubiquitin1 (*ubi1*) promoter. After transformation, 39 independent transgenic events were obtained from 360 bombarded IEs. The average transformation frequency (the total number of independent transgenic events divided by the total number of bombarded IEs) was 12.2% in these experiments. Transgenic events were confirmed by PCR screening at the T_0_ generation using gene-specific primers for the respective *SbPIN* genes and the *nptII* gene (Supplementary Table S1). More than 90% of transformants were fertile and displayed normal morphology when grown in pots in a containment glasshouse. The co-transformation rate of the *nptII* and *PIN* genes was 72% in these experiments. The segregation of *nptII* and target *PIN* genes in T_1_ progenies was observed with PCR screening and geneticin selection of seedlings, indicating that both were inherited in the T_1_ generation. The transformation procedure, from initiating IEs to planting putative transgenic plantlets in the glasshouse, was completed within 20 weeks.

### Genotypes in the T_1_ and T_2_ studies

The 17 lines in the T_1_ study comprised *SbPIN* transgenics, two negative PCR transgenics, plus a non-stay-green control (Tx430). Two of the transgenics (PIN1-5 and PIN1-12) were PCR negative for the gene of interest and hence were treated as a control (see section on statistical analysis of the T_1_ phenotyping study). Data from the four lines with the *ubi1* promotor driving *SbPIN2* expression are not presented here (hence data from only 10 transgenics are presented). The 10 transgenics assessed in this study consisted of two *SbPIN1* lines (PIN1-3 and PIN1-6), four *SbPIN2* lines (PIN2-1, PIN2-7, PIN2-12, and PIN2-14), and four *SbPIN4* lines (PIN4-3, PIN4-6, PIN4-8, and PIN4-11). T_1_ seeds from independent transformation events of PIN1-3, PIN1-6, PIN2-12, PIN2-14, PIN4-3, and PIN4-8 were used in the phenotypic and expression evaluations of the T_2_ generation, with Tx430 as the control. All plants were subjected to PCR screening using gene-specific primers for the respective *SbPIN* genes and the *nptII* gene (Supplementary Table S1; Supplementary Fig. S1).

### Growth conditions of T_1_ transgenics

Transgenic plants (T_1_) were grown in a PC2 glasshouse (recorded temperature range 18–28 °C) at The University of Queensland, Brisbane, Australia (27.50°S, 153.01°E). Experiments consisted of individual plants grown in 18 litre pots (330 mm diameter and 265 mm high). Each was filled with a 7:3 mix of composted pine bark (0–5 mm) and coco peat, and 30 g of Osmocote Plus (16% N, 3.5% P, 10% K plus trace elements; Scotts, Baulkham Hills, Australia) was added to each pot. The experiment was sown on 7 March 2017 (three seeds per pot) and all seedlings emerged 3 d later (10 March 2017). One week after emergence, seedlings were thinned to one per pot. The experiment was laid out as a randomized complete block design with seven replications and 17 lines randomized within each replicate. Plants were well watered and harvested soon after physiological maturity. Each pot was fertilized with 20 g of Osmocote at 10, 27 and 47 days after emergence (DAE). All plants were sprayed with a 0.07 M Ca(NO_3_)_2_ solution at 28, 31, and 34 DAE to prevent calcium deficiency. All-purpose soluble plant food (Miracle-Gro® 24% N, 3.5% P, 16% K with trace elements, Scotts Miracle Gro Inc., USA) was applied to each pot at 42, 49, 56, 63, and 70 DAE.

### Phenotyping of T_1_ transgenics

Anthesis was defined as the time when 50% of the anthers had extruded on the main panicle of each plant. Physiological maturity was defined as the time at which basal grains in 50% of the main panicles attained a black layer. The following shoot traits were phenotyped at the maturity harvest: height to the flag leaf, panicle base, and panicle top; panicle length; culm number (tillering); number of green mainstem leaves; area of all green mainstem leaves; area of tiller green leaves; length and width of FL–1 (flag leaf minus one); dry mass of the mainstem panicle, stem, green leaves, and dead leaves; dry mass of tiller panicles, stems, green leaves, and dead leaves; panicle number per plant; individual grain mass; and grain yield per plant. The following root traits were phenotyped at the maturity harvest: root length (maximum root depth); nodal root number; and root dry mass. For dry mass determination, each sample was dried in a forced draft oven at 80 °C for at least 48 h before weighing.

### Growth conditions of T_2_ transgenics

Transgenic plants (T_2_) were grown in a PC2 glasshouse (recorded temperature range 18–28 °C) at The University of Queensland, Brisbane, Australia (27.50°S, 153.01°E). T_1_ seeds from independent transformation events of PIN1-3, PIN1-6, PIN2-12, PIN2-14, PIN4-3, and PIN4-8 were used in the phenotypic evaluation of the T_2_ generation, with Tx430 as the control. Five seeds were sown per pot (3 cm depth) on 18 April 2019, and seedlings were thinned to one plant per pot ~1 week after emergence. All plants were sown in 18 litre ANOVApots® (Garden City Plastics, Dandenong South, Australia), with a total of seven biological replicates per genotype, laid out as a randomized block design. Each pot contained UQ23 potting medium (University of Queensland, St. Lucia, Australia) comprising 70% composted pine bark, 30% coco peat, and other additions. A 20 g aliquot of Osmocote Plus (16% N, 3.5% P, 10% K plus trace elements; Scotts, Baulkham Hills, Australia) was added to each pot at 10, 24, and 49 DAE. A 0.05 M solution of Ca(NO_3_)_2_ was applied to each plant at 7, 22, 28, 31, 35, 38, 42, and 46 DAE. Plants were watered daily and grown until physiological maturity.

### Phenotyping of T_2_ transgenics

A number of traits were phenotyped in the T_2_ generation to specifically align with T_2_ gene expression data. The following shoot and root traits that specifically related to the gene expression studies were phenotyped at the maturity harvest: culm number (tillering), total shoot dry mass per plant, length and width of FL–1 to FL–7, nodal root number per plant, and total root dry mass per plant. For dry mass determination, each sample was dried in a forced draft oven at 80 °C for at least 48 h before weighing.

### Derived variables

The harvest index was derived by dividing grain yield by above-ground dry mass. Grain number per plant was calculated by dividing grain yield per plant by the individual mass per grain. The linear stem density of the mainstem was calculated by dividing the mass of the mainstem by its length. Total plant green leaf area at maturity was the sum of mainstem green leaf area and tiller green leaf area. Total plant dry mass was the sum of mainstem dry mass and tiller dry mass. Leaf appearance rate (LAR) was derived by dividing mainstem leaf number by the estimated number of days to flag leaf emergence, where the date of full flag leaf appearance was assumed to have occurred 12 d before flowering. An ‘assimilate demand index’ was created by multiplying LAR by the average size of mainstem leaves (ranging from leaves FL–3 to FL–7).

### Statistical analysis of the T_1_ phenotyping study

The 126 pot trial was laid out as a row–column array with 21 columns and six rows. The experimental design was a randomized complete block design with seven replicates of 17 lines (119 pots) plus one missing plot (seven pots) laid out as blocks containing six rows and three columns (totalling 126 pots). The 17 lines were comprised of 14 transgenics, two negative PCR transgenics, plus the control. A one-way ANOVA of the two negative PCR lines showed that the negative PCR lines were not significantly different from the control, Tx430. These negative PCR lines were combined to create a single control. Data from the four lines with the *ubi1* promotor driving *SbPIN2* expression are not presented here (hence data from 10 lines only are presented). All traits were analysed using a linear mixed model with a fixed line effect and random spatial effects that accounted for the pot position in the trial plus a random residual effect. A Wald test was used to test for significance between lines using a χ^2^ statistic with 14 degrees of freedom. The predicted line effects were each tested against the control using a normal two-tailed test for significance.

### Statistical analysis of the T_2_ phenotyping study

The experimental design was a randomized block design with seven replicates of seven lines (49 pots), with each block located on a separate bench. Statistical comparisons were analysed using one-way ANOVA followed by Dunnett’s multiple comparisons test using GraphPad Prism version 9.0.2 for Windows (GraphPad Software, San Diego, CA, USA).

### Expression studies [quantitative reverse transcription–PCR (RT–qPCR)]

In order to establish the role of the *SbPIN* genes in generating the phenotypes described above, and their role in modulating plant architecture and hence stay-green, we analysed the expression of *SbPIN1* (PIN1-3), *SbPIN2* (PIN2-12, PIN2-14), and *SbPIN4* (PIN4-3, PIN4-8) genes at 28 (early tillering) and 35 (floral initiation) DAE in four tissue types (leaf blade, leaf sheath, meristem, and root) of the T_2_ transgenics relative to the control.

We sampled at the times and in the specific organs where we would predict the greatest developmental impact based on information from two databases (Phytozome and MOROKOSHI). Our main reference for sampling of tissues and the selected time points came from transcriptome data of BTx623 (McCormick *et al.*, 2018). MOROKOSHI was the original RNA-seq database that we used to identify tissues of *SbPIN1*, *SbPIN2*, and *SbPIN4* that would be expressed. This database has been updated and now also includes McCormick’s data (Makita *et al.*, 2015). A previous study (Shen *et al.*, 2010) also showed the expression of *SbPIN* genes in leaf, flower, stem, and root in 3-week-old sorghum plants. In McCormick’s study, they sampled leaf, leaf sheath, shoot (including shoot meristem and stems), and root at stages they termed vegetative (24 DAE) and floral initiation (44 DAE). We would expect the *PIN* genes to be expressed in tissues associated with the causal mechanisms of stay-green, namely meristem tissue associated with tillering; leaf blade and sheath tissue associated with leaf size; and root tissue associated with root architecture. Therefore, we measured the expression of *SbPIN* genes in four tissue types (leaf blade, leaf sheath, meristem, and root) at 28 (early tillering) and 35 (floral initiation) DAE.

Whereas all 10 transgenics assessed in this study were phenotyped, only five of the transgenics (PIN1-3, PIN2-12, PIN2-14, PIN4-3, and PIN4-8) were used in the gene expression studies. These five transgenics exhibited the full range of phenotypic variation and were thus representative of the range of shoot and root phenotypes displayed by the 10 transgenics (e.g. high versus low tillering, small versus large organ size). Sampling was undertaken at the early tillering and floral initiation stages because of the likely impact of auxin on branching and panicle meristem development at these stages, respectively. Seeds from T_1_ transgenics (lines) PIN1-3, PIN2-12, PIN2-14, PIN4-3, and PIN4-8, and the non-stay-green control (Tx430) were planted in 18 litre ANOVApots® (Garden City Plastics) at three biological replicates per pot, per harvest time point. Each pot contained UQ23 potting medium (University of Queensland, St. Lucia, Australia) comprising 70% composted pine bark and 30% coco peat and other additions. A 30 g aliquot of Osmocote Plus (16% N, 3.5% P, 10% K plus trace elements; Scotts) was added to each pot. Whole leaf blade and leaf sheath of the fifth leaf, 1 cm of the leaf whorl section containing the apical meristem, and nodal roots were harvested at 28 and 35 DAE. All tissues harvested were immediately placed into liquid nitrogen and stored at –80 °C prior to RNA extraction. Total RNAs were extracted using the ISOLATE II RNA Plant Kit (Bioline), with on-column DNase treatment, according to the manufacturer’s protocol. cDNA synthesis was carried out with 50 ng of RNA using the GoScript™ Reverse Transcription System (Promega). RT–qPCR was performed using GoTaq® qPCR Master Mix (Promega) on a CFX384 Touch Real-Time PCR Detection System (Bio-Rad), using ubiquitin-conjugating enzyme (*UbCE*; Sb09g023560) (Shakoor *et al.*, 2014; Kumar *et al.*, 2020) as the reference gene. Relative gene expression was measured and calculated between control (Tx430) and transgenic lines across three biological replicates with three technical replicates using the comparative 2^−ΔΔCt^ method (Livak and Schmittgen, 2001). Statistical analysis was performed using two-way ANOVA followed by Tukey’s multiple comparisons test utilizing GraphPad Prism version 9.0.2 for Windows (GraphPad Software). In addition, relative expression of *SbPIN4* in the transgenic lines of PIN4-3 and PIN4-8 were also evaluated against another reference gene: RNA recognition motif-containing protein (*RRM*; Sb07g027950) (Shakoor *et al.*, 2014; Kumar *et al.*, 2020). The relative expression of *SbPIN4* in all tissues and time points tested was not statistically different between the reference genes used (Supplementary Fig. S2). All primer sequences are listed in Supplementary Table S1. Expression of various *SbPIN* genes in various organs at various time points (T_2_ experiment) was plotted against the relevant T_2_ phenotypic data to determine the impact of gene expression on phenotype.

### *Modelling of the effects of* PIN *genes*

The measured relative effects of PIN 2-14 (up-regulated for *SbPIN2* in the leaf blade at 35 DAE and down-regulated for *SbPIN2* in the meristem at 28 DAE) on leaf size distribution and tillering were incorporated in the APSIM simulation model (version 7.10, revision 4176) (Holzworth *et al.*, 2014) and its sorghum module (Hammer *et al.*, 2010) parameterized for commonly used genotypes in Australia (Buster—triple-dwarf grain hybrid) and India (M35-1—single dwarf dual-purpose inbred line). Simulations were conducted for the control (without PIN 2-14 effects) and the modified PIN types in both environments. For both situations, a 30% decrease in the size of the largest leaf was invoked (in line with the measured response from whole-plant studies in a contained glasshouse). This generated a leaf size–leaf number distribution with smaller leaves in a similar fashion to that observed with PIN 2-14 (Supplementary Fig. S3). In addition, for Buster (a high-tillering type), the normal level of tillering (2.1 fertile tillers per plant) was reduced (to 1.47 fertile tillers per plant), in line with the measured response from whole-plant studies in a contained glasshouse. As M35-1 is low tillering in its normal production system, simulations assumed no tillers for both the control and modified type. For control genotype parameterizations and model validation, see Hammer *et al.* (2010). Simulations were conducted for Buster (and Buster-PIN) at Dalby, Australia, using historical weather data for the period 1969–2018. For each year, the simulated crop was planted on 15 October, assuming a standard local agronomy of 5 plant m^–2^ on 1 m rows. A local vertosol soil was assumed with 140 cm depth and plant available water-holding capacity (PAWC) of 240 mm (APSoil number: 14). It was assumed that the profile was half full (120 mm available water) at the time of planting. For M35-1 (and M35-1-PIN), simulations were conducted at Solapur, India, using historical weather data for the period 1977–1997. For each year, the simulated crop was planted on 10 October, assuming a standard local agronomy of 10 plants m^–2^ with 0.5 m row spacing. A local vertosol soil was assumed with 90 cm depth and PAWC of 145 mm. It was assumed that the profile was full at the time of planting at the beginning of the Rabi season after the monsoon season. In both situations, simulations were conducted presuming non-limiting nutrient supply. One individual season with characteristic terminal stress per location was selected to show simulated leaf area development, soil water, crop biomass, and grain yields, and summaries of yield effects over the entire time series were prepared.

## Results and discussion

### *Genes from the* PIN *family in sorghum co-locate with stay-green QTL*

**Fig. 2.**
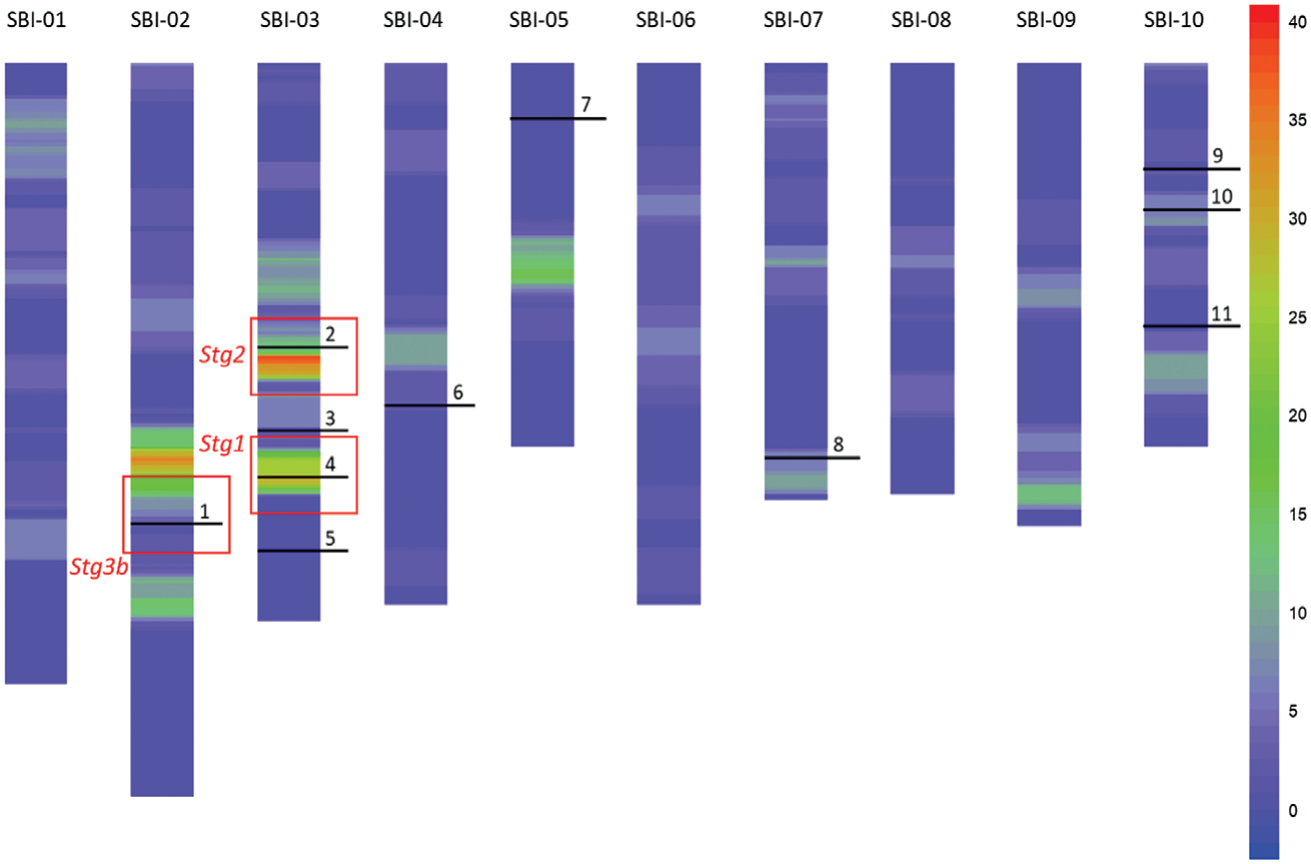
Co-location of *SbPIN* genes with stay-green QTL. Stay-green QTL density plots demonstrating the location of the 11 *SbPIN* genes (indicated as a horizontal line and numbered 1–11). Scale bar to the right indicates QTL density per 2 cM. Nine of the 11 genes from the *SbPIN* family in sorghum co-located with *Stg* QTL. *SbPIN5*, located on chromosome 3, and *SbPIN7*, located on chromosome 5, were the only *SbPIN* genes that did not co-locate with *Stg* QTL. The *Stg1*, *Stg2*, and *Stg3b* QTLs (indicated as red boxes) contain *SbPIN4*, *SbPIN2*, and *SbPIN1*, respectively, highlighting the key stay-green regions studied herein.

We found that nine of the 11 members of the *PIN* family of auxin efflux carriers in sorghum co-locate with *Stg* QTL (Fig. 2). In fact, *SbPIN4* and *SbPIN2* co-located with two of the major *Stg* QTL (*Stg1* and *Stg2*, respectively) that have been used in detailed studies into the physiological basis of stay-green in sorghum (Borrell *et al.*, 2014a, b). Furthermore, *SbPIN1* co-located with the *Stg3b* QTL, a key region under selection in the Australian public sector sorghum breeding programme that has a strong focus on yield improvement in water-limited environments (Potgieter *et al.*, 2016). *PIN* genes have been associated with plant architecture traits in other cereals such as rice, where *OsPIN1* and *OsPIN2* affect tiller number (Xu *et al.*, 2005; Chen *et al.*, 2012), whereas *OsPIN1* and *OsPIN3t* have been associated with adventitious root emergence (Xu *et al.*, 2005; Zhang *et al.*, 2012). While the effects of *OsPIN3t* on adventitious root growth have been linked to drought stress tolerance in rice (Zhang *et al.*, 2012), *PIN* genes have not previously been identified as important elements in the molecular mechanism underpinning the stay-green drought adaptation trait.

### PIN *genes affect canopy size*

We transformed three *PIN* genes, *SbPIN1*, *SbPIN2*, and *SbPIN4* with their native promoters, in a non-stay-green (senescent) sorghum (Tx430) and examined their effects on canopy architecture traits. Canopy size in cereals is affected by the number of culms (tillering), the number of leaves per culm, and the size of individual leaves. The *SbPIN* genes, to varying degrees, affected all of the aforementioned canopy traits. However, some of these effects are likely to be pleiotropic, either through allometric relationships or through internal competition for assimilates. The number of tillers, for example, may be a consequence of a primary effect on mainstem leaf size. Demand for assimilates increases with increasing organ size, such that plants with good early vigour of the main shoot (i.e. large leaves and high LAR) have less excess assimilate availability for tiller bud outgrowth than plants with small leaves or low LAR (Alam *et al.*, 2014).

**Fig. 3.**
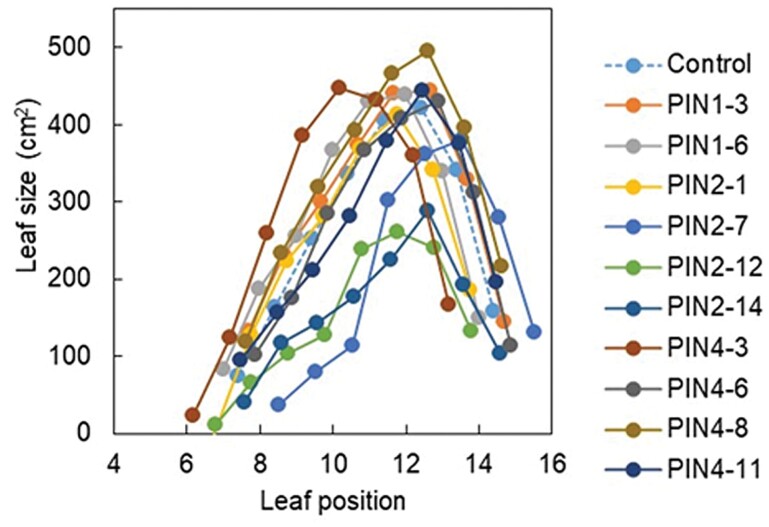
*SbPIN* genes modify leaf size in sorghum. Leaf size distributions of control (dashed line) and various overexpression lines (solid lines, PIN transgenics) grown in the T_1_ phenotyping study.

The PIN transgenics differed significantly in leaf size (Fig. 3), with PIN2-12 (*P*<0.001) and PIN2-14 (*P*<0.001) having small mainstem leaves, whereas PIN4-3 [non-significant (ns)] and PIN4-8 (*P*=0.07) tended to have the largest mainstem leaves. The link between gene expression in the leaf blade at 35 DAE and leaf length is discussed later (see Fig. 9A and C). Leaf appearance rate ranged from 0.232 (PIN4-3) to 0.285 (PIN1-3) leaves per day, with the control exhibiting a LAR of 0.275 leaves per day (data not shown).

**Fig. 4.**
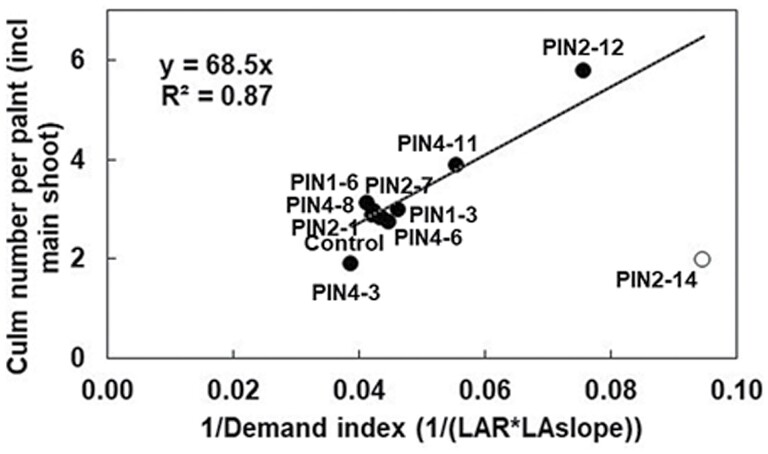
Differences in tillering are explained by assimilate demand or *PIN* genes in a set of sorghum PIN transgenics and the control. Culm number per plant versus the inverse of carbon demand, which is represented by the product of leaf appearance rate (LAR) and the slope of increase in leaf area for successive fully expanded leaves (LAslope, estimated from leaves seven to three below the flag leaf). Culm number per plant includes the main stem. Open symbols were excluded from the regression, which was forced through the origin. Data were collected from plants grown in the T_1_ phenotyping study.

The PIN transgenics also affected branching (tillering). In the T_1_ study, culm number per plant significantly differed from the control and increased in the following order: PIN2-14 (1.98 culms per plant, *P*<0.05)<Tx430 (2.83)<PIN2-12 (5.79, *P*<0.01). Similar results were obtained in the T_2_ study, with culm number per plant differing as follows: PIN2-14 (2.5, *P*<0.05)<Tx430 (4.5)<PIN2-12 (12, *P*<0.01). As a measure of assimilate demand by the mainstem, we created an assimilate demand index by multiplying LAR by the average rate of increase in size of mainstem leaves positioned seven to three leaves below the flag leaf and plotted total culm number (tillers plus mainstem) against the inverse of this index (Fig. 4). For most PIN transgenics, total culm number was close to that of the control, which was consistent with a similarity in demand index (Fig. 4). For PIN4-3 (low tillering), PIN4-11 (medium tillering), and PIN2-12 (high tillering), the difference in tillering compared with the control could largely be explained as a consequence of differences in assimilate demand. In contrast, PIN2-14 had a much lower culm number per plant than was expected based on its assimilate demand index (Fig. 4), indicating that decreased bud outgrowth was likely to be due to altered *PIN* expression. This will be explored in more detail in the section on ‘Transgenics overexpressing *SbPIN2*’ below. In summary, the *PIN* genes probably regulate branching both indirectly (via an effect on main shoot organ size which in turn impacts the availability of excess assimilate for bud outgrowth) and directly (via suppression of bud outgrowth due to altered *PIN* expression).

The observation that *PIN* genes can reduce mainstem leaf size, combined with their effects to reduce tillering, means that they can decrease pre-anthesis crop water demand (Borrell *et al.*, 2014a; George-Jaeggli *et al.*, 2017). This preserves water in the subsoil that will allow increased water uptake during the grain-filling period, which can result in the retention of green leaf area to support prolonged crop growth (Fig. 1), characteristic of a stay-green phenotype. These emergent consequences are further explored in the section below on ‘Modelling shows that *SbPIN2* phenotype can increase grain yield under drought’.

### PIN *genes affect root architecture*

Roots enable plants to access water and nutrients, as well as providing anchorage. The distribution of roots in the soil through space and time, known as root architecture, is the result of three cellular processes: curving, elongating, and branching (Rich and Watt, 2013). Importantly, we hypothesize that root architecture is regulated by a combination of allometric and hormonal responses, with *PIN* genes probably acting indirectly via allometry and directly via hormones (i.e. a direct response to altered *PIN* expression). Allometric responses include the indirect above-ground effects of *PIN* genes on below-ground root architecture (i.e. root number and mass and, to a lesser extent, length). An indirect effect indicates that *PIN* genes primarily impact above-ground processes such as branching (through either direct or indirect effects on axillary bud outgrowth) and, as a consequence of allometry, impact root traits (e.g. number and mass). We hypothesize that the effects of *PIN* genes on root architecture that cannot be explained by allometric relationships are likely to be under hormonal control (i.e. a direct effect of *PIN* genes via hormones).

**Fig. 5.**
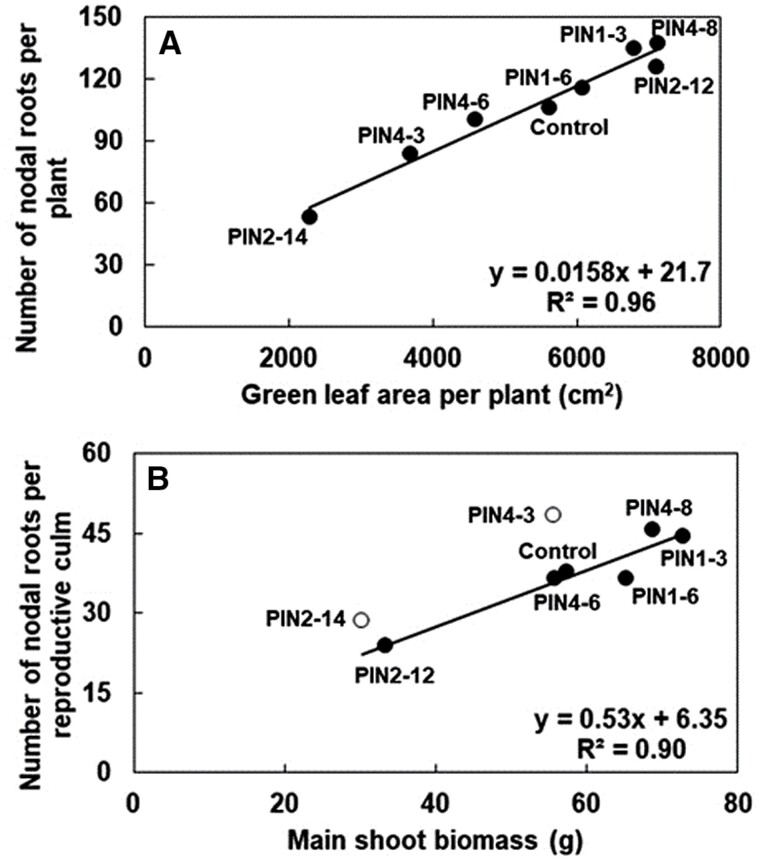
*SbPIN* genes modify above- and below-ground growth in a set of sorghum PIN transgenics. (A) Nodal root number per plant versus green leaf area per plant. (B) Number of nodal roots per reproductive culm (defined as a culm with a fertile panicle) versus main shoot biomass. Note that each of these graphs contains only eight data points (not 11 as in the other figures) since root data were only collected on a subset of eight genotypes. Filled symbols sit on the regression line; open symbols are outliers. Data were collected from plants grown in the T_1_ phenotyping study.

The effects of *PIN* genes on roots appeared to be largely allometric with shoot characteristics, as above-ground plant size, measured as maximum leaf area per plant (largely a consequence of above-ground branching), was positively correlated with the number of nodal roots per plant (*r*^2^=0.96, *n*=8, Fig. 5A) and with root mass per plant (*r*^2^=0.93, *n*=8). Despite this strong association of nodal root traits with shoot biomass on a ‘per plant’ basis, it is worth noting that some transgenics (e.g. PIN4-3) exhibited high nodal root number per reproductive culm (defined as a culm with a fertile panicle), despite low nodal root numbers per plant (Fig. 5). The *SbPIN* transgenics varied almost 3-fold in nodal root number per plant, a branching trait, ranging from 53 nodal roots (PIN2-14, *P*<0.01, Fig. 5A) to 137 (PIN4-8, *P*<0.05, Fig. 5A). The control produced 106 nodal roots (Fig. 5A). The number of nodal roots per reproductive culm generally increased with dry mass of the main shoot (Fig. 5B), indicating that plants with larger culms produced more nodal roots per reproductive culm (based on the assumption that nodal root number per culm is equivalent for the mainstem and tillers).

However, two transgenics (PIN2-14 and PIN4-3) had slightly higher nodal root number per reproductive culm relative to their main shoot biomass (Fig. 5B). PIN2-14 and PIN4-3 were also the transgenics with low (*P*<0.05) tiller numbers (Fig. 4), although these genotypes exhibited low tillering for different reasons—PIN4-3 had a high demand index which resulted in low tillering, whereas PIN2-14 had a low demand index—suggesting that low tillering was regulated by something other than assimilate demand (e.g. hormones). This could imply that, at least for PIN2-14, the relatively high nodal root number per reproductive culm was potentially a consequence of excess assimilate availability in response to low demand for tiller production (i.e. an indirect effect of *PIN* genes). Plotting root expression of *SbPIN2* at 28 DAE versus total root biomass suggests that *SbPIN2* may also have a more direct role in root architecture (see the section on ‘Transgenics overexpressing *SbPIN2*’). The possible role of the *PIN* genes in regulating root architecture will be discussed later.

Sorghum plants transformed with *SbPIN* genes also exhibited significant (*P*<0.01) variation in root length (measured as the length of the longest root), ranging from 449 mm (PIN4-3) to 748 mm (PIN1-6). The control had a maximum root length of 633 mm.

### PIN *genes affect panicle growth and grain yield*

**Fig. 6.**
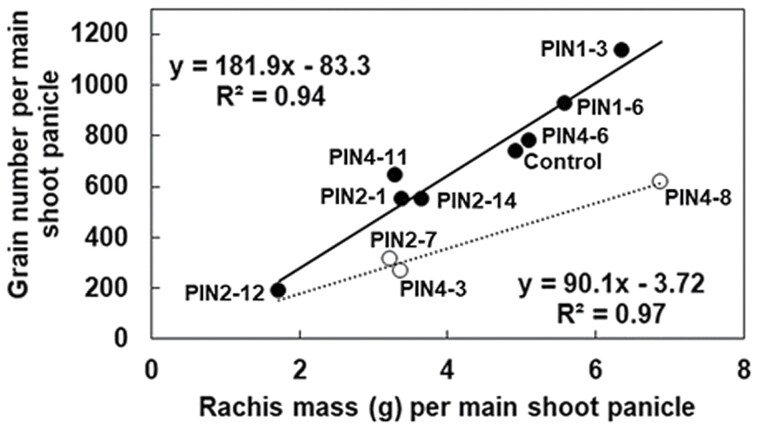
Allometric relationship between grain number and rachis mass of the main shoot panicle. The main regression line is denoted by the solid line and filled symbols. The alternative regression line is denoted by a dashed line and open symbols. Data were collected from plants grown in the T_1_ phenotyping study.

The PIN transgenics varied >5-fold in grain yield per plant, ranging from 13 g to 67 g per plant, compared with 43 g per plant in the control. Grain yield per plant was predominantly determined by grain number per plant (*r*^2^=0.90, *n*=11), rather than by individual grain mass (*r*^2^=0.25, *n*=11). Much of the variation in grain number was associated with plant size, since for most PIN transgenics, grain number of the main shoot was linearly associated with its rachis mass (Fig. 6). Grain yield per plant was 56% greater (*P*<0.05) in PIN1-3 compared with the control. PIN1-3 had the highest grain number per main shoot panicle, PIN2-12 the lowest, and grain number increased by ~180 for each gram increase in rachis mass (Fig. 6). Similar results have been reported in rice (except that down-regulation rather than overexpression of *PIN* genes enhanced grain yield). Transgenic rice plants containing an RNAi vector to knock-down endogenous transcripts of the *OsPIN5b* gene exhibited longer panicles, and more seeds per panicle and per plant, than the wild type, resulting in ~30% more grain yield (Lu *et al.*, 2015). However, three of the transgenics in the current study, PIN2-7, PIN4-3, and PIN4-8, only had half the number of grains that would have been expected based on rachis mass (Fig. 6). This could indicate a potentially adverse effect of some *PIN* genes on reproductive processes. Similarly, *OsPIN5b* overexpression lines in rice exhibited reduced seed set, shorter panicles, and fewer full seeds per panicle and per plant, thereby resulting in lower yield compared with the wild type (Lu *et al.*, 2015). Overall, the higher grain yields relative to Tx430 of PIN1-3, and to a lesser extent of PIN1-6, were largely explained by the allometric relationship between grain number and rachis mass of the main shoot panicle (Fig. 6). However, differences in rachis mass (the causal mechanism for enhanced grain number) are possibly due to altered *PIN* expression (see the section on ‘Transgenics overexpressing *SbPIN1*’).

### *Effects of* PIN *genes on organ size*

**Fig. 7.**
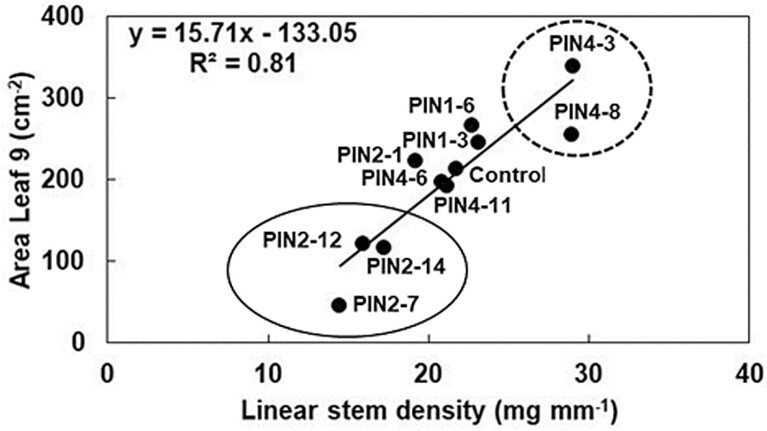
Allometric relationship between linear stem density (LSD) and the area of leaf 9 (LA9) in a set of sorghum PIN transgenics. Three transgenics with low values for LA9 and LSD are all PIN2 (solid ellipse) and two transgenics with high values for LA9 and LSD are both PIN4 (dashed ellipse). Data were collected from plants grown in the T_1_ phenotyping study.

Individual *SbPIN* genes had contrasting effects on leaf area and organ size. *SbPIN2* transformations generally resulted in smaller vegetative organs, as the PIN2-12 and PIN2-14 transgenics consistently exhibited small leaf size (Figs 3, 7) and low linear stem density (Fig. 7). As a consequence, PIN2-12 and PIN2-14 transgenics also had a low carbon demand (high index, Fig. 4). PIN4-3 and PIN4-8, on the other hand, consistently had large organs, as was evident from large leaf area (Figs 3, 7), high linear stem density (Fig. 7), and high rachis mass (PIN4-8 only, Fig. 6). Lines that overexpressed *SbPIN1* generally exhibited larger (*P*<0.05) reproductive organs (mainstem rachis mass, grain yield per plant; Fig. 6) compared with the control. Furthermore, total above-ground biomass (*P*<0.05), nodal root number per plant (*P*<0.05, Fig. 5A), and total biomass of roots plus shoot (*P*<0.05) were also higher in PIN1-3 than in the control, with a trend for these traits to be higher in PIN1-6. This was despite PIN1-3 and PIN1-6 exhibiting culm numbers per plant comparable with the control (Fig. 4). These effects on yield components may partly explain why the *Stg3b* QTL, which harbours *SbPIN1*, has been under selection in the Australian public sector sorghum breeding programme. Overall, individual transgenics within each group of *PIN* genes could have contrasting effects on selected traits.

### PIN *genes differentially expressed in various organs relative to non-stay-green control*

Expression of *PIN* genes from a stay-green source in a non-stay-green genotype resulted in differential expression in various tissues and phenotypes that was consistent with the causal effects of the stay-green trait. This result can be interpreted as showing: (i) that modulation of *PIN* expression can mimic the causal mechanisms driving stay-green phenotypes (this is of practical utility), and possibly (ii) that differences in *PIN* gene expression or function are the molecular basis of stay-green versus non-stay-green phenotypes in the corresponding genotypes.

**Fig. 8.**
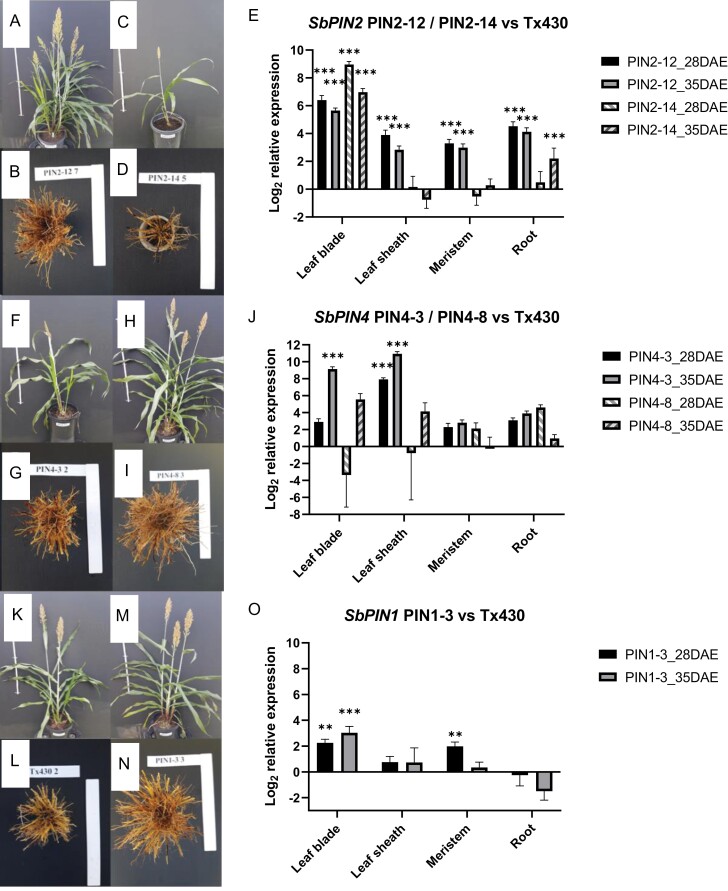
Phenotypes and gene expression of *SbPIN* transgenics compared with the control (Tx430). Above- and below-ground phenotypes of PIN2-12 (A and B) and PIN2-14 (C and D). Tissue-specific *SbPIN2* expression patterns of PIN2-12 and PIN2-14 relative to the control (Tx430) at 28 and 35 DAE (E) in the leaf blade (LB), leaf sheath (LS), meristem (M), and root (R). Above- and below-ground phenotypes of PIN4-3 (F and G) and PIN4-8 (H and I). Tissue-specific *SbPIN4* expression patterns of PIN4-3 and PIN4-8 relative to the control at 28 and 35 DAE (J) in the LB, LS, M, and R. Above- and below-ground phenotypes of the control (Tx430) (K and L) and PIN1-3 (M and N). Tissue-specific *SbPIN1* expression patterns of PIN1-3 relative to the control at 28 and 35 DAE (O) in the LB, LS, M, and R. Phenotypic and expression data are from the T_1_ and T_2_ studies, respectively.

**Fig. 9.**
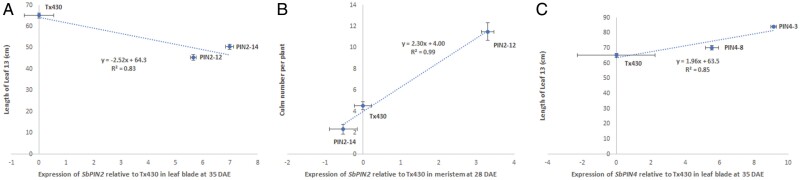
Expression of *PIN* genes correlated with phenotype. (A) Expression of *SbPIN2* in the leaf blade at 35 DAE versus length of leaf 13 (FL–1), *P*<0.01, (B) expression of *SbPIN2* in the meristem at 28 DAE versus culm number per plant, *P*<0.001, and (*C*) expression of *SbPIN4* in leaf sheath at 35 DAE versus length of leaf 13 (FL–1), *P*<0.01. Phenotypic and expression data were both collected from the T_2_ study.

Crucially, we were able to identify clear differences in *PIN* gene expression among independent transgenics, ranging from little change in expression in some tissues to many-fold overexpression. The target genes were overexpressed in the transgenics, and the effects were in the organs we expected from the stay-green trait (leaves, tillers, and roots) and at the time points we expected (early tillering and floral initiation). Transcript analysis revealed that as well as the expected higher *PIN* gene expression through the ‘dosage effect’, there were a number of independent transgenics with high copy number of the inserted genes, but little change in expression in some tissues, indicating the variability of ‘position effect’ of the inserted transgene (Peach and Velten, 1991; Meyer, 1995). We demonstrated that the tissue-specific expressions of *SbPIN2* in the T_2_ transgenics could explain the modulation in tillering and leaf size in the phenotypic studies of T_1_ and T_2_ transgenics (Figs 8, 9), and that the tissue-specific expression of *SbPIN4* in the T_2_ transgenics could explain the modulation in leaf size in the phenotypic studies of T_1_ and T_2_ transgenics (Figs 8, 9). The possible roles of *SbPIN1* in panicle architecture and *SbPIN2* in root architecture are also discussed.

Plasticity of adaptive plant development in response to environmental changes can be attributed, to a large extent, to dynamic changes in PIN transporters of the plant hormone auxin (Grunewald and Friml, 2010). Different aspects of auxin distribution-mediated development, including gravitropism, phototropism, embryogenesis, organogenesis, vascular tissue formation, and regeneration, are modulated by the polar, plasma membrane localization of PIN proteins which determine the direction of auxin flow within tissues (Vanneste and Friml, 2009). Based on our data, we argue that changes in the expression of the *SbPIN* genes in the relevant tissues (i.e. leaf blade, leaf sheath, meristem, and root) and at specific times (i.e. early tillering and floral initiation) have directly modified canopy and root characteristics that are associated with transpiration and water uptake (i.e. tillering, leaf size, and root architecture) driving the stay-green phenotype in sorghum.

### *Transgenics overexpressing* SbPIN2

Transgenics overexpressing *SbPIN2* exhibited contrasting phenotypes at both the above- and the below-ground levels (Fig. 8A–D), including a very low tillering phenotype probably caused by perturbation of gene expression in the meristem (PIN2-14, Fig. 8C, D). *SbPIN2* was expressed differentially in the PIN2-14 (low-tillering, small leaf size phenotype) and PIN2-12 (high-tillering, small leaf size phenotype) lines across three of the four tissue types (except leaf blade tissue) at 28 (early tillering) and 35 (floral initiation) DAE (Fig. 8E). The up-regulated (*P*<0.05) expression of PIN2-12 and PIN2-14 in the leaf blade and of PIN2-12 in the leaf sheath at both sampling times (Fig. 8E) corresponded to their small leaf size (as measured in the five uppermost leaves) relative to the control in the transgenic phenotypic study (Fig. 3); for example, the individual areas of FL–1 to FL–5 were significantly (*P*<0.05) smaller in PIN2-12 (Fig. 8A) and PIN2-14 (Fig. 8C) compared with the control (Fig. 8K). Expression of *SbPIN2* in the leaf blade at 35 DAE (T_2_) was highly negatively correlated (*r*^2^=0.83, *P*<0.01, *n*=3) with the length of leaf 13 (T_2_), suggesting that the increased expression of *SbPIN2* in that organ at that time point decreased leaf length (Fig. 9A).

Localized regulation of auxin, via differences in *SbPIN2* expression, would be expected to affect processes including organ primordia positioning, stem cell maintenance, fruit patterning, and the ability of auxin to regulate cell division, expansion, and differentiation (Salehin *et al.*, 2015). We hypothesize that higher *SbPIN2* expression induces transport of indole-3-acetic acid (IAA) from leaves (specifically from the leaf basal growing zone), resulting in smaller leaves by reducing IAA levels that are needed to sustain leaf growth, thereby reducing the rate or duration of leaf growth.

At 28 DAE, *SbPIN2* expression was up-regulated (*P*<0.001) in the meristem of PIN2-12 relative to the control, whereas *SbPIN2* expression was down-regulated (ns) in the meristem of PIN2-14 (Fig. 8E). The differences in *SbPIN2* expression in the meristem between PIN2-14 and PIN2-12 at early tillering (28 DAE) relative to the control corresponded to the differences observed for tillering propensity in the phenotypic study (Fig. 4). In the T_1_ study, culm number per plant was significantly higher (*P*<0.01) in PIN2-12 (5.79, Fig. 8A) compared with the control (2.83, Fig. 8K), which was significantly higher (*P*<0.05) than in PIN2-14 (1.98, Fig. 8C). In the T_2_ study, culm number per plant was significantly higher (*P*<0.01) in PIN2-12 (12.0) compared with the control (4.5), which was significantly higher (*P*<0.05) than in PIN2-14 (2.5). Expression of *SbPIN2* in the meristem at 28 DAE (T_2_) was highly positively correlated (*r*^2^=0.99, *P*<0.001, *n*=3) with culm number per plant (T_2_), suggesting that the increased expression of *SbPIN2* in that organ at that time point increased tillering (Fig. 9B). This increase in tillering with increased relative expression of *SbPIN2* in the meristem at early tillering would indicate that, conversely, the trend towards down-regulation of *SbPIN2* in the meristem at 28 DAE could confer decreased plant canopy size via a reduced number of tillers. Such differences in tillering propensity that are not a consequence of differences in carbon demand (e.g. PIN 2-14 in Fig. 4) are likely to be under hormonal control (Alam *et al.*, 2014), and a direct response to altered *PIN* expression. Auxin is a primary candidate via *PIN* gene action, but the role of other hormones (e.g. strigolactones) should not be excluded, since strigolactones can repress the gene expression and protein accumulation of *PIN* auxin transporters (Crawford *et al.*, 2010; Brewer *et al.*, 2013).

At both sampling times, *SbPIN2* expression was higher (*P*<0.001 at 28 DAE and 35 DAE) in the roots of PIN2-12 compared with PIN2-14 (ns at 28 DAE and *P*<0.001 at 35 DAE) (Fig. 8E), corresponding somewhat to the differences observed in the phenotypic study for root mass (24.7 versus 7.4 g per plant, respectively, T_1_, *P*=0.06; 62.5 versus 12.4 g per plant, respectively, T_2_, *P*<0.01) and nodal root numbers per plant (53 versus 16, respectively, T_1_, *P*<0.01, Fig. 8B, D; 190 versus 70, respectively, T_2_, *P*<0.001). Furthermore, expression of *SbPIN2* in the roots at 28 DAE (T_2_) was correlated (*r*^2^=0.81, *P*<0.05, *n*=3) with total root biomass (T_2_), suggesting that the increased expression of *SbPIN2* in that organ at that time point may be associated with the increase in root mass observed. The differences in nodal root number, at least, may be due to allometric relationships (Fig. 5A) rather than (or in addition to) the direct effect of *SbPIN2* overexpression. Previous studies in maize have found that overexpression of *ZmPIN1a* elevated IAA concentrations in roots, increasing the number of lateral roots (Li *et al.*, 2018).

At 35 DAE, compared with 28 DAE, overall *SbPIN2* expression was decreased in all tissues in both PIN2-14 and PIN2-12, except in the meristem and root of PIN2-14 (Fig. 8E). Early expression of *SbPIN2* prior to, or at 28 DAE, is likely to be critical for determining plant architecture via mechanisms such as tillering and leaf size, and possibly via root mechanisms (i.e. root mass and nodal root numbers).

### *Transgenics overexpressing* SbPIN4

There were no significant differences from Tx430 in expression levels in any tissues for PIN4-8 (Fig. 8F–J). However, PIN4-3 showed significant overexpression in leaf blades and leaf sheaths at 35 DAE (floral initiation, *P*<0.001, Fig. 8J), and in leaf sheaths at 28 DAE (early tillering, *P*<0.001, Fig. 8J), and the phenotype exhibited larger (ns, T_1_; *P*<0.001, T_2_) leaves (FL–3 to F–5, Fig. 3). Leaf blade expression of *SbPIN4* at 35 DAE (T_2_) was highly positively correlated (*r*^2^=0.85, *P*<0.01, *n*=3) with length of leaf 13 (T_2_), suggesting that the increased expression of *SbPIN4* in that organ at that time point increased leaf length (Fig. 9C).

PIN4-3 exhibited a trend for lower nodal root number and root mass, and significantly (*P*<0.01) shorter root length compared with the control (Fig. 8GL). The ‘higher than expected’ number of nodal roots per reproductive culm for a given main shoot biomass indicates that something, in addition to allometry, might be driving root number in PIN4-3 (Fig. 5B). The expression (ns) of *SbPIN4* in the roots at 35 DAE may be linked to these changes in phenotype. However, the observation that these root phenotypes were not accompanied by clear and significant differences in expression levels in the root tissue could suggest that they are more likely to be caused indirectly by allometric relationships (Fig. 5A), rather than by a direct response to altered *PIN* expression.

### *Transgenics overexpressing* SbPIN1

PIN1-3 showed significant overexpression in leaf blades at 28 DAE (early tillering, *P*<0.01, Fig. 8O) and 35 DAE (floral initiation, *P*<0.001, Fig. 8O). However, there were no significant differences in leaf size distribution between PIN1-3 and Tx430 (Fig. 3), indicating that the overexpression of *SbPIN1* was not likely to be associated with leaf size in this case, but potential associations with other leaf traits cannot be ruled out. Transgenics overexpressing *SbPIN1* exhibited increased rachis mass (*P*<0.05; Fig. 6), panicle mass (*P*<0.05), grain number per panicle (*P*<0.1; Fig. 6), and grain yield (*P*<0.05) compared with the control (Fig. 8K, M).The overexpression of *SbPIN1* in the meristem at 28 DAE (*P*<0.01) could be a driver of these phenotypic differences. Nodal root number was significantly (*P*<0.05) greater in PIN1-3 relative to the control (135 versus 106, Fig. 8L, N), although this may have been a consequence of differences in culm number (Fig. 5A), suggesting that the higher nodal root number in PIN1-3 was due to allometric relationships rather than a direct response to altered *PIN* expression (Fig. 5B). The lack of significant differences in *SbPIN1* expression compared with the control in the roots at either 28 or 35 DAE supports this conclusion.

### Expression in tissues associated with the causal mechanisms of stay-green

Although the stay-green phenotype is only apparent after anthesis, it is largely an emergent consequence of changes to the transpirational leaf area during vegetative growth and root characteristics. We would expect the *PIN* genes to be expressed in tissues associated with canopy characteristics which have previously been shown to lead to the development of a stay-green phenotype when water is limiting after flowering, namely meristem tissue associated with tillering, leaf blade and sheath tissue associated with leaf size, and root tissue associated with root architecture. However, the tillering, leaf size, and root architecture responses are largely constitutive; that is, these responses occur early in crop growth (and generally before drought is a problem). For example, down-regulation of *SbPIN2* in the meristem leads to low tillering, and up-regulation in the leaf blade leads to small leaves early in crop growth, resulting in decreased water demand prior to flowering (water savings), and hence increased water availability during grain filling (utilizing the water savings). Hence, expression data under well-watered conditions and at the times and in the tissues in which we expect these changes to occur (as presented herein) seem appropriate for these constitutive mechanisms, the emergent consequence of which is a drought adaptation phenotype.

### PIN *genes affect transpirational leaf area and root growth in a way that improves water availability during the critical grain-filling period*

The stay-green phenotype is an emergent consequence of the interaction between *Stg* loci that regulate largely constitutive traits related to plant size (i.e. tillering and leaf size), and hence water demand by the crop, and the environment that regulates water supply by the soil. Hence, these constitutive traits, regulated by *PIN* genes, set the plant up to evade drought stress during grain filling due to the comparatively higher plant available soil moisture.

### Connecting across scales

Connecting molecular understanding to whole-plant physiology is required to scale-up from gene level to the farmer’s field. We have previously shown that delayed leaf senescence during grain filling (functional stay-green) is an emergent consequence of dynamics occurring earlier in crop growth (e.g. smaller canopy size at flowering) and is largely due to an improved balance between the supply and demand of water after flowering (Borrell *et al.*, 2014a, b), which we hypothesize is primarily mediated by the effects of key *PIN* genes on canopy development and root architecture. Other approaches such as transcriptomics could also shed light on the molecular understanding of stay-green.

### *Modelling shows that the* SbPIN2 *phenotype can increase grain yield under drought*

Genetic (G) and management (M) solutions are required to develop resilient crops in highly variable environments (E). Many different management systems are possible to combat drought (e.g. combinations of planting dates, fertilizers, irrigation, row spacing, population, and cropping systems). Many different genotypic solutions are also possible, including utilizing the *PIN* gene family. The challenge is to identify favourable combinations of genotypes and management practices in a complex system. Understanding the interaction between genotypes, management, and the environment (G×M×E) is critical to improving grain yield under dry conditions.

Strategies that specifically target genetic and management solutions for adaptation to drought can be evaluated using simulation modelling, enabling plant breeders and grain growers to ask informed questions such as: ‘What type of canopy development would work best in my current environments and management systems?’ We used crop simulation modelling (APSIM-sorghum model; Hammer *et al.*, 2010) to assess the impact of the *SbPIN2* gene on canopy development (leaf size and tillering) in sorghum. Modelling using data from end-of-season drought stress environments in Australia and India showed that the reductions in leaf size and tillering associated with the *SbPIN2* gene effect generated reduced canopy size, a slower rate of water use/soil water decline, and a stay-green phenotype.

Overall, crop simulation modelling demonstrated that the effects of down-regulating *SbPIN2* in the meristem (i.e. decreasing tillering) coupled with up-regulation in the leaf blade (i.e. decreasing the size of leaves) reduces canopy size and increases grain yield under end-of-season drought in Australia and India. The effects of up- or down-regulation of *SbPIN2* on tillering and leaf size (in line with the measured response in a Tx430 background from whole-plant studies in a glasshouse) were captured in APSIM by reducing tillering and leaf size in Buster PIN and M35-1 PIN compared with the default parameters of these genotypes. Hence, the model does incorporate the expected physiological changes (e.g. tillering and leaf size in this case) that have been shown to be caused by up- or down-regulation of the gene of interest, although it does not operate directly at the molecular level.

**Fig. 10.**
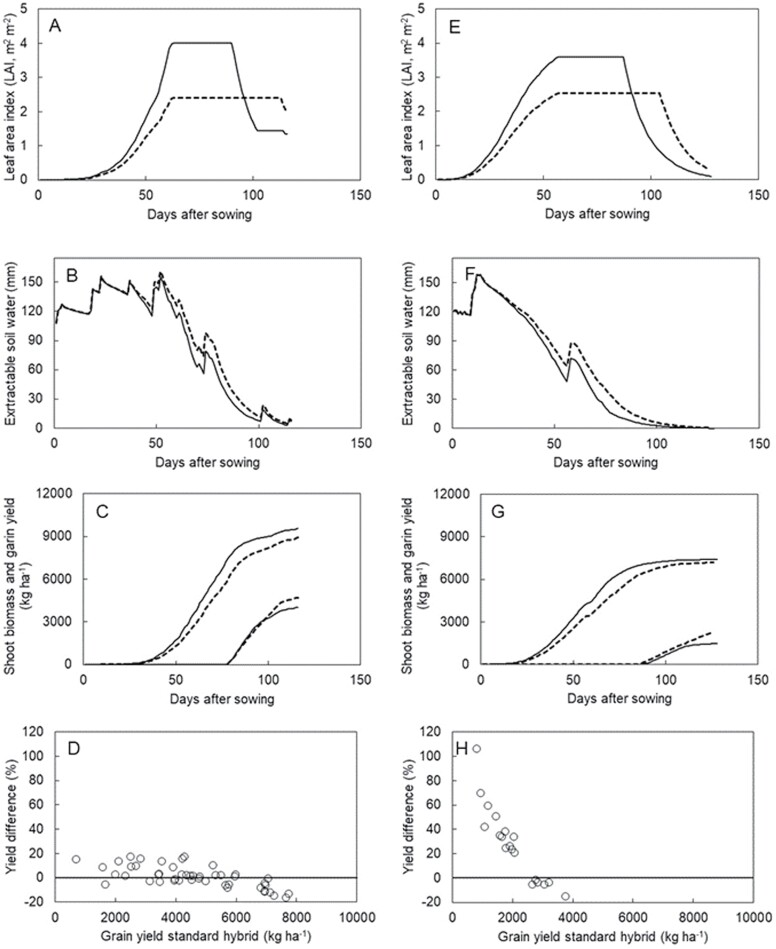
Modelling the impacts of *PIN* genes on leaf area, soil water, biomass, and risk. The emergent consequences of reduced canopy size (i.e. smaller leaves and lower tillering) due to down-regulation of *SbPIN2* in the meristem (i.e. reduced tillering) and up-regulation in the leaf blade (i.e. smaller leaves) is shown for a single year with characteristic post-anthesis water stress in Australia for the senescent hybrid Buster (A–C) and in India for the senescent cultivar M35-1 (E–G) with the *PIN* gene effect (dotted line) and without the *PIN* gene effect (solid line) in terms of temporal leaf area development (A, E), extractable soil water (B, F), and biomass and grain yield (C, G). Risk scenarios highlighting yield advantages of the presence of the PIN gene relative to the yield of the standard hybrid for multi-year simulations of Buster grown at Dalby in Australia (D) and M35-1 grown at Solapur in India (H). Note that there were different numbers of years used for simulations in Australia (~50) and India (~20) due to weather data availability.

The generation of stay-green as an emergent property of the reduced canopy size and associated water saving is evident in the single-year simulations for 1978 at Dalby, Australia, and 1993 at Solapur, India, which were chosen to reflect the common end-of-season stress environments experienced in these production environments. The reduced leaf area of the *SbPIN2* type (Buster PIN, Dalby, Fig. 10A; M35-1 PIN, Solapur, Fig. 10E) caused slower use of available water (Fig. 10B, F), enabling longer retention of green leaf area and enhanced growth during grain filling, leading to greater yield of the *PIN* type (Fig. 10C, G). Significant yield advantage was associated with the *SbPIN2* gene effects in nearly all years at Solapur (Fig. 10H). In this environment (Indian *Rabi* season), nearly all years will experience an end-of-season stress situation (Kholová *et al.*, 2013). Yield advantages of 0.5–1.0 t ha^–1^ were frequent in crops yielding <2.5 t ha^–1^.

A similar outcome was found at Dalby in Australia, although the greater range in seasonal rainfall resulted in a greater range of responses. Significant yield advantage occurred in most situations where the control (Buster) yielded <6 t ha^–1^ (Fig. 10D). However, some negative effects of the *SbPIN2* gene on yield were simulated in wet seasons where yield of the control exceeded 6 t ha^–1^. At the crop level, it should be possible to overcome these negative effects by optimizing G×M combinations (e.g. increasing crop density of *PIN* types in wetter seasons). It is possible to have phenotypic attributes such as stay-green as emergent properties of the model dynamics by enhancing the explanatory power of the modelling approach without undue complexity (Hammer *et al.*, 2016). This approach should enable the linking of genotype to phenotype and hence, molecular biology and genetics with crop improvement.

### Conclusions

Cereals are highly sensitive to water stress during grain filling. We used sorghum as a model crop to understand how the *PIN* genes may regulate causal mechanisms of the stay-green drought adaptation phenotype in cereals. For crops grown on soils with a moderate to high water-holding capacity, any mechanism that constrains pre-anthesis water demand, increases water supply, or enhances water use efficiency (other things being equal) should increase post-anthesis biomass production and grain yield when water is a limiting factor. The *PIN* genes are one such mechanism. Our results demonstrate that modulation of *PIN* expression can mimic the causal mechanisms driving stay-green phenotypes and, possibly, that differences in *PIN* gene expression or function is the basis of stay-green versus non-stay-green phenotypes in the corresponding genotypes.

In some of the associations shown here, the evidence linking gene expression to phenotype is more compelling than in others. Of the more compelling evidence, expression of *SbPIN2* in the meristem at 28 DAE probably regulates tillering, and expression of *SbPIN2* and *SbPIN4* individually in the leaf blade at 35 DAE is likely to regulate leaf size. Evidence suggesting that expression of *SbPIN2* in the roots at 28 DAE regulates root biomass is also relatively strong. The evidence that components of panicle architecture may also be regulated by the expression of *SbPIN1* at 28 DAE is less compelling, since only one transgenic (PIN1-3) was compared with the control. Hence further studies are warranted to clarify the impact of *SbPIN* genes on root and panicle architecture.

We also acknowledge that we have not presented either cell biological or biochemical documentation of *PIN* localization or alterations in auxin transport consistent with functional changes in *PIN* activity, and that our results need to be considered within this context. Further studies are warranted to address these issues. It is hypothesized that the mechanisms by which the *PIN* genes regulate canopy development, and possibly root architecture and grain yield, also function similarly in the world’s other major cereal crops to enhance productivity under drought. The next step is to evaluate the impact of *PIN* genes on canopy development, root growth, and panicle architecture in maize, rice, wheat, and barley, ultimately determining whether these genes do confer drought adaptation at the field scale in these cereals.

## Supplementary data

The following supplementary data are available at *JXB* online.

Fig. S1. Gene-specific primers (for *SbPIN1*, *SbPIN2*, and *SbPIN4*) and primers targeting the *nptII* gene were used for PCR screening of transgenic plants.

Fig. S2. Log_2_ relative expression of *SbPIN4* in the tissues tested for PIN4-3 and PIN4-8 at 28 and 35 DAE using ubiquitin-conjugating enzyme and RNA recognition motif-containing protein as reference genes.

Fig. S3. Simulations of reduced leaf size due to down-regulation of *SbPIN2* for commonly used genotypes in Australia (Buster; AB) and India (M35-1; CD).

Table S1. List of primers and sequences for gene targets.

erac336_suppl_supplementary_figures_S1-S3_table_S1

## Data Availability

All data supporting the findings of this study are available within the paper and within its supplementary data published online.
