# Supplementary material for: Genetic modification of PIN genes induces causal mechanisms of stay-green drought adaptation phenotype
Source: J Exp Bot. 2022 Aug 13;73(19):6711–26. doi: 10.1093/jxb/erac336 (PMC9629789; doi:10.1093/jxb/erac336)
Supplement: erac336_suppl_supplementary_figures_S1-S3_table_S1 [file erac336_suppl_supplementary_figures_s1-s3_table_s1.pdf]

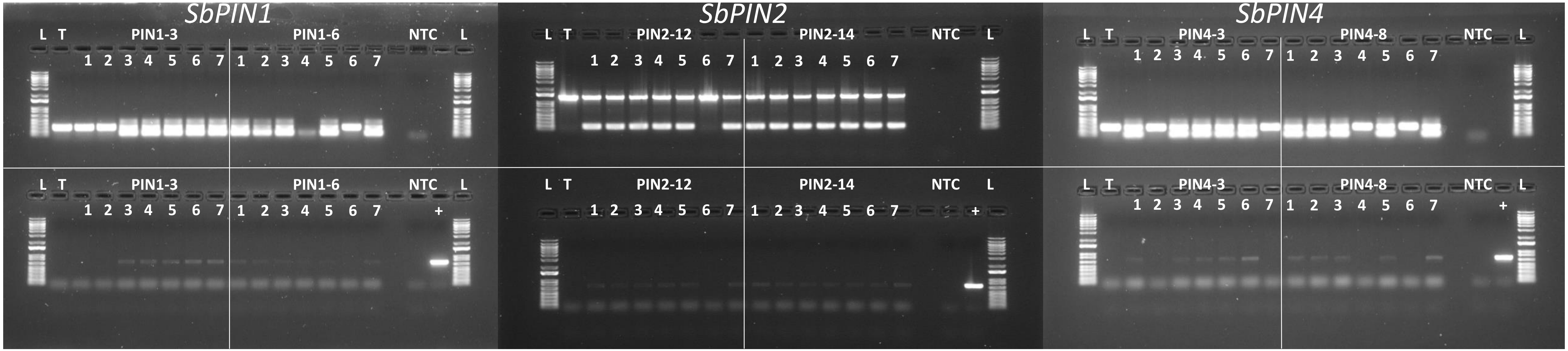

Fig. S1. Gene-specific primers (for *SbPIN1*, *SbPIN2* and *SbPIN4*) and primers targeting the *nptII* gene were used for PCR screening of transgenic plants. Primers for *SbPIN* genes were designed to span an intron in each of the target genes. (Top row) For *SbPIN1*, transgenics contained product sizes of 125bp and 272bp, and non-transgenics contained only a product size of 272bp. For *SbPIN2*, transgenics contained product sizes of 155bp and 1115bp, and non-transgenics contained only a product size of 1115bp. For *SbPIN4*, transgenics contained product sizes of 100bp and 264bp, and non-transgenics contained only a product size of 264bp. (Bottom row) For *nptII*, transgenics contained a product size of 740bp, and non-transgenics showed no product amplification. “L” indicates 1 Kb Plus DNA Ladder (Invitrogen) . “T” indicates Tx430 control. “NTC” indicates no template control. “+” indicates positive control using diluted *nptII*-containing plasmid.

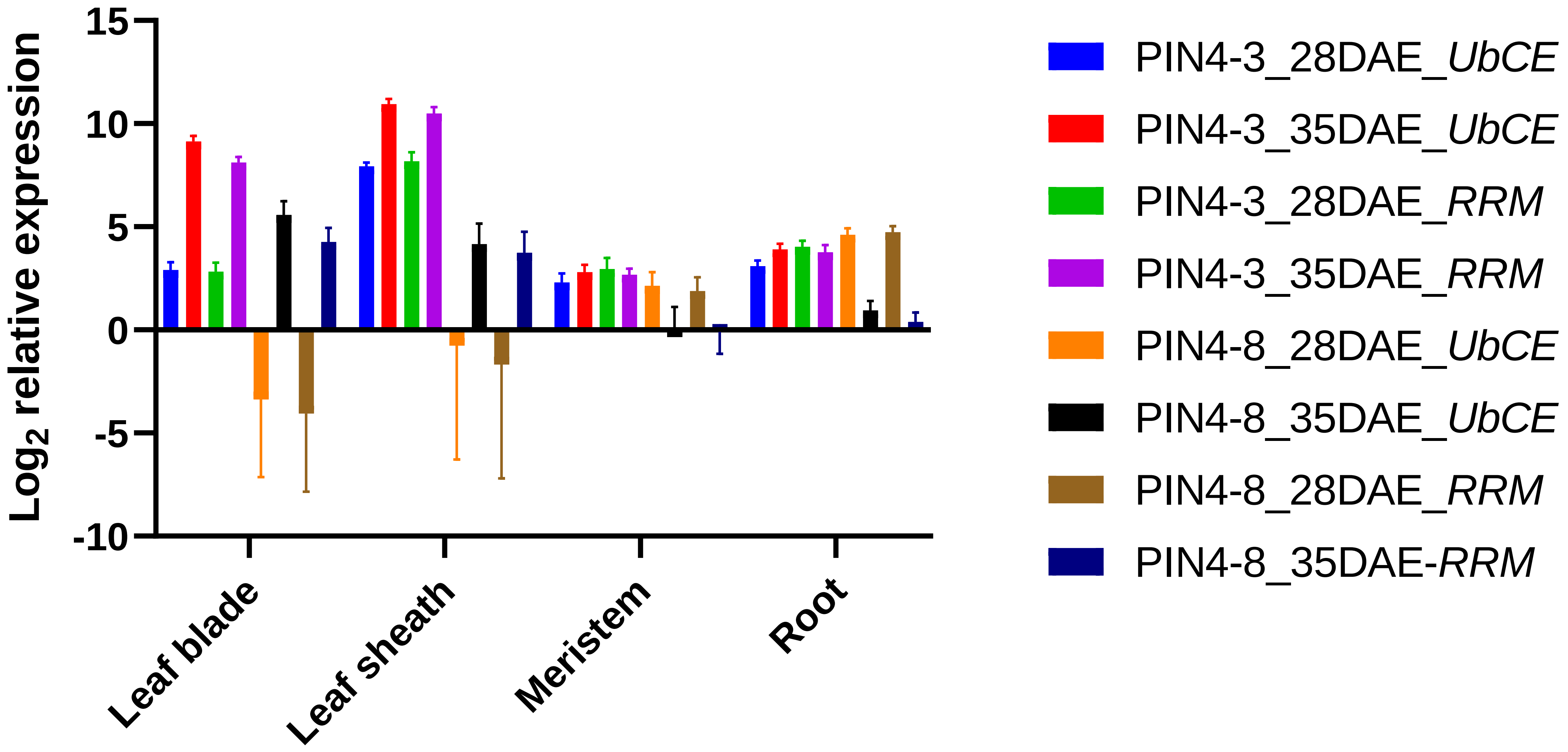

15
10
5
0
-5
-10
Log2 relative expression
Leaf blade
Leaf sheath
Meristem
Root
PIN4-3_28DAE_UbCE
PIN4-3_35DAE_UbCE
PIN4-3_28DAE_RRM
PIN4-3_35DAE_RRM
PIN4-8_28DAE_UbCE
PIN4-8_35DAE_UbCE
PIN4-8_28DAE_RRM
PIN4-8_35DAE-RRM

Fig. S2. Log_2 relative expression of *SbPIN4* in the tissues tested for PIN4-3 and PIN4-8 at 28 DAE and 35 DAE, using ubiquitin-conjugating enzyme (*UbCE*) and RNA recognition motif-containing protein (*RRM*) as reference genes. Two-way ANOVA analyses show that *SbPIN4* expression levels in all tissues of PIN4-3 and PIN4-8 tested at both sampling time points are not statistically different between the reference genes used. The error bars represent standard deviation (n=3).

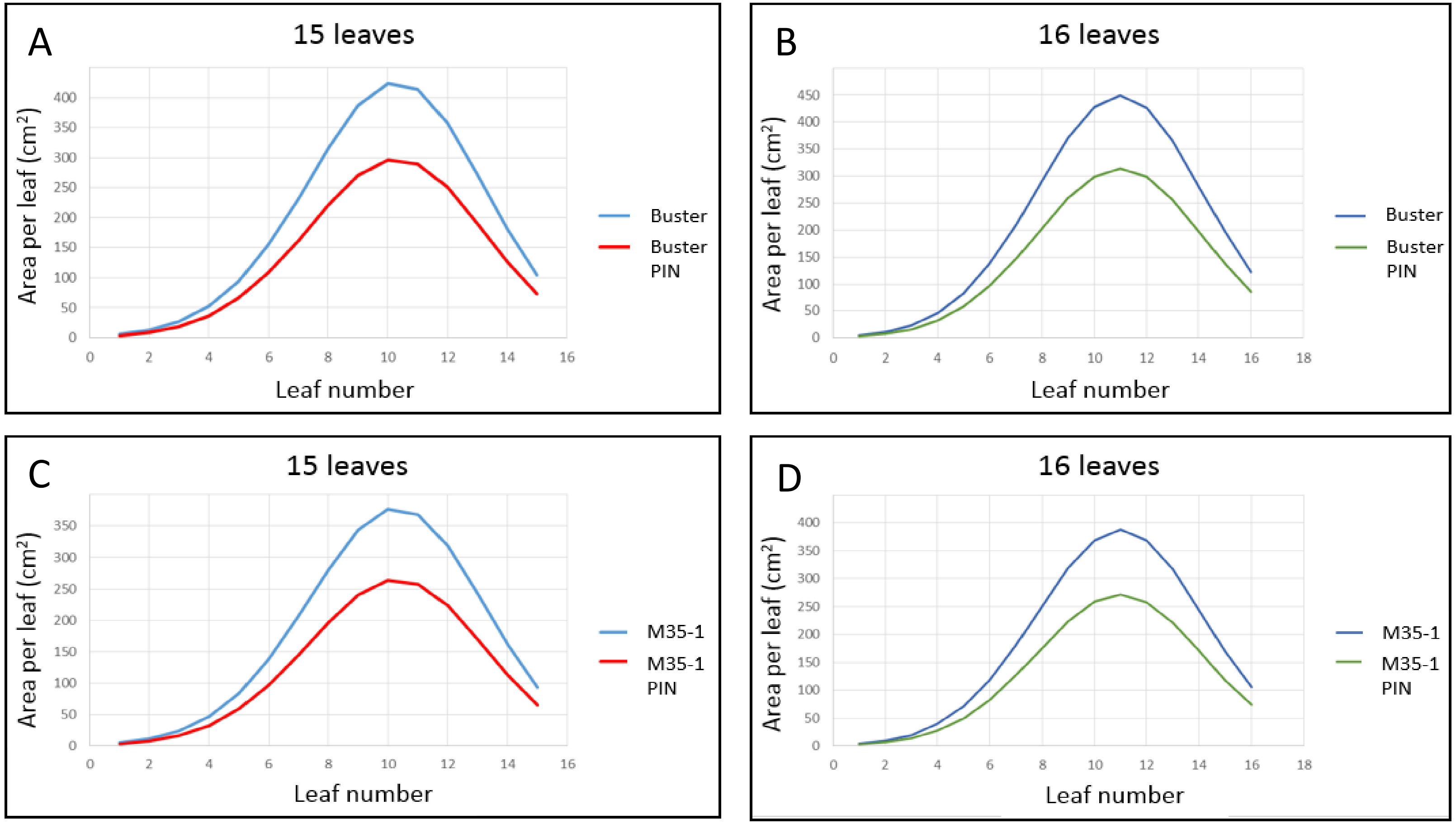

Fig. S3. Simulations of reduced leaf size due to down-regulation of *SbPIN2*. The measured relative effects of PIN 2-14 (down-regulated for *SbPIN2*) on leaf size distribution were incorporated in the APSIM-sorghum model parameterised for commonly used genotypes in Australia (Buster – triple-dwarf grain hybrid) for plants with (*A*) 15 leaves and (*B*) 16 leaves, and India (M35-1 – single dwarf dual-purpose inbred line) with (*C*) 15 leaves and (*D*) 16 leaves. Simulations were conducted for the control (without PIN 2-14 effects) and the modified type in both environments. For both situations, a 30% decrease in the size of the largest leaf was invoked (in line with measured response). This generated a leaf size-leaf number distribution with smaller leaves in a similar fashion to that observed with PIN 2-14.

Table S1. Primers list

Gene / Target	Primer	5'-3' Sequence	Tm
SbPIN1	RTP1F8	CGTTCGTGTTCGCCAGGG	60°C
	RTP1R8	ACACCGTATCAAGCCTAGAACAAT	60°C
SbPIN2	RTP2F4	AACACGTACTCCAGCCTCATC	60°C
	RTP2R4	TGCAGCGCCATAAACAATCCTA	60°C
SbPIN4	RTP4F5	CCTTGGCCTCATCTGGTCTC	60°C
	RTP4R5	CCCAACTGCGGTTGTGTAGA	60°C
sorghum ubiquitin-conjugating enzyme UbCE, Sb09g023560	Ref_Ubi_F	CGACCAGCAACAAACCCAAG	60°C
	Ref_Ubi_R	CCCTGAGATTGCCCACATGT	60°C
RNA recognition motif containing protein (RRM; Sb07g027950)	RRM-RT-F	CTGAACGAGCGTTGTCGTTG	60°C
	RRM-RT-R	AGCACGCCCTCTAAAGGAAC	60°C
nptII	KAN-F	AGACAATCGGCTGCTCTGAT	59.98^{o}C
	KAN-R	TCATTTCGAACCCCAGAGTC	60.05^{o}C
